# Gallium nanoparticles facilitate phagosome maturation and inhibit growth of virulent *Mycobacterium tuberculosis* in macrophages

**DOI:** 10.1371/journal.pone.0177987

**Published:** 2017-05-18

**Authors:** Seoung-ryoung Choi, Bradley E. Britigan, David M. Moran, Prabagaran Narayanasamy

**Affiliations:** 1 Department of Pathology and Microbiology, College of Medicine, University of Nebraska Medical Center, Omaha, Nebraska, United States of America; 2 Department of Internal Medicine, College of Medicine, University of Nebraska Medical Center, Omaha, Nebraska, United States of America; 3 Research Service, Veterans Affairs Medical Center-Nebraska Western Iowa, Omaha, Nebraska, United States of America; Institut de Pharmacologie et de Biologie Structurale, FRANCE

## Abstract

New treatments and novel drugs are required to counter the growing problem of drug-resistant strains of *Mycobacterium tuberculosis* (*M*.*tb*). Our approach against drug resistant *M*.*tb*, as well as other intracellular pathogens, is by targeted drug delivery using nanoformulations of drugs already in use, as well as drugs in development. Among the latter are gallium (III) (Ga)-based compounds. In the current work, six different types of Ga and rifampin nanoparticles were prepared in such a way as to enhance targeting of *M*.*tb* infected-macrophages. They were then tested for their ability to inhibit growth of a fully pathogenic strain (H37Rv) or a non-pathogenic strain (H37Ra) of *M*.*tb*. Encapsulating Ga in folate- or mannose-conjugated block copolymers provided sustained Ga release for 15 days and significantly inhibited *M*.*tb* growth in human monocyte-derived macrophages. Nanoformulations with dendrimers encapsulating Ga or rifampin also showed promising anti-tuberculous activity. The nanoparticles co-localized with *M*.*tb* containing phagosomes, as measured by detection of mature cathepsin D (34 kDa, lysosomal hydrogenase). They also promoted maturation of the phagosome, which would be expected to increase macrophage-mediated killing of the organism. Delivery of Ga or rifampin in the form of nanoparticles to macrophages offers a promising approach for the development of new therapeutic anti-tuberculous drugs.

## Introduction

In 2015, it was estimated that 9 million people developed tuberculosis (TB) due to infection with *Mycobacterium tuberculosis (M*.*tb)* and 1.5 million died from TB infection according to the World Health Organization (WHO).[1] The widespread emergence of multi-drug resistant *M*.*tb* and extensively drug resistant *M*.*tb* poses a serious threat to public health, making the development of new antibacterial drugs to treat TB of great importance.[2]

A key component of TB pathogenesis is that the causative bacilli survive, grow and replicate within host macrophages. This is due in part to their ability to inhibit the maturation of the phagosome by blocking phagosome fusion with lysosomes to form the phagolysosome, an acidic and hydrolytic compartment that is microbicidal.[3] The successful parasitization of macrophages is an ingenious way through which *M*.*tb* avoids the immune response of host cells. [4–11]

Iron, an essential nutrient for nearly all living cells, plays a critical role in many important enzymatic reactions as a cofactor. Its ability to redox cycle between Fe(II)/Fe(III) enhances electron transfer.[12] In humans, iron is tightly bound to transferrin, lactoferrin, ferritin and heme. Pathogenic bacteria must acquire iron mainly from these iron complexing proteins for growth and metabolism. Many pathogens possess highly efficient iron uptake mechanisms. These bacteria release iron solubilizing (chelating) compounds, siderophores, to obtain Fe^3+^ from host iron-binding molecules for growth. Carboxymycobactin and mycobactin are mycobacterial siderophores that chelate Fe^3+^ extracellularly and intracellularly, and are critical to the virulence of these organisms in vitro and in vivo.[13–17]

Since *M*.*tb* resides in macrophage and their growth is dependent on iron acquisition and utilization, targeting *M*.*tb* iron acquisition within host macrophages could be a promising strategy for treatment of *M*.*tb* infection. [13–17]

Gallium (Ga), a group IIIA metal, is similar to iron. Gallium is known to interfere with iron acquisition by microorganisms.[16–19] Although iron and Ga have similar chemical properties, Ga(III) cannot be reduced to Ga(II). Hence, if iron is replaced with Ga in biologically important proteins that participate in cell metabolism, these functions are inhibited. To date, antimicrobial activity of Ga compounds have been explored and demonstrated against many pathogenic bacteria, including *M*.*tb*.[16–19] Also, it has been reported that gallium nitrate inhibits biofilm formation and growth of *Pseudomonas aeruginosa*.[18]

We recently confirmed that blocking iron acquisition in *M*.*tb* is a potential way to reduce the growth of intracellular *M*.*tb*.[20] Interference of iron acquisition was achieved using antimicrobial Ga (III) nanoparticles. Most recently, we reported that targeting macrophages with a long acting Ga nanoformulation that produced sustained release of Ga (III) resulted in growth inhibition of *Mycobacterium smegmatis* and *M*.*tb*.[21] This strategy was also previously applied to develop a macrophage-targeted nanoparticle containing conventional antiretroviral therapy to inhibit virus replication.[20–22]

The above results further supported the potential of Ga nanoparticles for the treatment of virulent *M*.*tb*. The present work is intended to further develop that potential through the creation of nanoparticles that should more selectively target the nanoparticles to *M*.*tb*-infected macrophages, thereby enhancing the therapeutic index. We prepared 6 differently ligated Ga(III) nanoparticles with poloxamer F127 or dendrimer. The resulting nanoparticles had different size, morphology, ζ-potential and drug content. The characterized nanoparticles were tested against the growth of virulent *M*.*tb* (H37Rv) in macrophages. In addition, given the importance of phagolysosome fusion to macrophage-mediated killing of *M*.*tb*, we investigated the effect of Ga(III) nanoparticles on the process of phagosome maturation.

## Materials and methods

### Materials

All chemicals were purchased from Sigma-Aldrich unless otherwise stated. Gallium(III) *meso* tetraphenylporphyrine chloride (GaTP) was purchased from Frontier Scientific (Logan, Utah, USA). NMR spectroscopies were recorded on a Varian Unity/Inova-500 NB (500 MHz, Varian Medical Systems Inc. Palo Alto, CA, USA). Chemical shifts are reported in parts per million (ppm) downfield from TMS. A Hitachi S4700 Field-Emission Scanning electron microscope (SEM, Hitachi High Technologies America, Inc., Schaumburg, IL, USA) was used for images of nanoparticles. Confocal images were obtained using a Zeiss LSM 510 or 710 laser scanning microscope (Carl Zeiss, Inc. Thornwood, NY. USA).

### Synthesis of mannose-conjugated F127

Succinic anhydride (0.88 mmol) was added slowly at room temperature (rt) to a solution of F127 (MW ~12,500 Da, 5 g, 0.4 mmol), dimethyl amino pyridine (DMAP, 0.4 mmol) and triethyl amine (TEA, 0.8 mmol) in CH_2_Cl_2_. The mixture was stirred at rt for 24 h and the solvent was removed under reduced pressure. The remaining crude products were precipitated by adding cold diethyl ether while stirring in an ice bath. The precipitates were collected, washed with ether 3 times and dried under vacuum to afford a white powder (P407-COOH, 4.8 g, 91%). The product was analyzed using ^1^H-NMR (S1 and S2 Figs).

P407-COOH was activated with N, N’-dicyclohexylcarbodiimide (DCC) and N-hydroxy succinimide (NHS) in CH_2_Cl_2_ at rt for 24 h. NHS (5 eq) was added to a solution of F127-COOH (0.047 mmol, MW ~12600 Da) and DCC (5 eq) in CH_2_Cl_2_ at rt. After stirring for 24 h, D-mannose (5 eq) and TEA (5 eq) in DMSO was added to the reaction mixture and stirred at rt for 24 h. The urea byproduct was filtered off, and then CH_2_Cl_2_ was evaporated under reduced pressure. The remaining crude products were precipitated by addition of cold ether while stirring. The white powder was collected, washed with ether and dried (40%). The product was analyzed using ^1^H-NMR (S1 and S2 Figs).

### Synthesis of folate-conjugate F127

Folic acid (220mg, 0.5 mmol) and F127 (3.1g, 0.25 mmol) was refluxed for 4 h in presence of 23 mL of ammonium hydroxide. The obtained product was washed with water, dried and characterized by ^1^H-NMR (S1 and S2 Figs).

### Preparation and characterization of nanoparticles containing Ga(III) or rifampin

Nanoparticles containing Ga(III) or rifampin were formulated and manufactured using high pressure homogenizer (Avestin C5 high-pressure homogenizer, Avestin Inc, Ottawa, ON, USA) and characterized by dynamic light scattering (DLS, a Malvern Zetasizer Nano Series Nano-ZS, Malvern Instruments Inc., Westborough, MA, USA) and SEM, as reported before.[20]

### Cytotoxicity assay

1 × 10^5^ THP-1 cells/well were placed in a 96-well plate and differentiated in RPMI 1640 containing 10% fetal serum, PMA (7.5 ng/mL), 10 mM sodium pyruvate, 50 μg/mL gentamicin and 10 mM HEPES (pH 7.0) at 37°C in 5% CO_2_ humidified atmosphere for 24. After washing with PBS buffer, the THP-1 macrophages were treated with nanoparticles (100, 300, and 500 μM) suspended in RPMI 1640 containing 10% fetal serum for 24 h. The cells were washed with PBS three times. Fresh media (100 μL) was then added to each well and their ability to reduce resazurin was measured.

### Measurement of drug uptake by THP-1 cells

THP-1 cells (7.5 × 10^5^ per well) were differentiated in RPMI 1640 containing 10% fetal serum, PMA (7.5 ng/mL), 10 mM sodium pyruvate, 50 μg/mL gentamicin and 10 mM HEPES (pH 7.0) at 37°C in 5% CO_2_ humidified atmosphere for 24. After washing with PBS buffer twice, THP-1 cells were treated with 300 μM nanoparticles in RPMI 1640 media for 24 h. Uptake of nanoparticles by THP-1 cells were determined at 1, 4, 8 and 24 h incubation. Adherent cells were washed with PBS buffer three times and collected by scraping into 1.5 mL of PBS at the designated incubation time. The cells were centrifuged at 14,000 × g for 10 min and the supernatants were discarded. Cell pellets were sonicated in 200 μL of methanol and centrifuged at 21000 × g at 4°C for 15 min. Drug amounts were determined by ICP-MS for Ga(III) or HPLC/UV spectroscopy for rifampin. Drug retentions in THP-1 cells were assessed at day 1, 5, 10 and 15 following nanoparticle treatment for 24 h. THP-1 cells were washed, collected, centrifuged, and drug amounts were determined as mentioned above.

### Analysis of Ga(III) by ICP-MS

Gallium was measured on a Perkin Elmer NexION 300D inductively-coupled mass spectrometer (Perkin Elmer, Shelton, CT). Briefly, solutions were diluted 1:100 in 2% nitric acid and mixed. From this 50 μL were added to 3.65 mL of a solution of 1% nitric acid, 3 mM EDTA, 0.03% Triton-X100^®^, and 10 ppb terbium (Tb). The Tb was used as an internal standard. A five-point calibration curve was generated, against which the samples were compared for quantitation. The raw response of Ga from each sample was divided by the raw response for Tb and this ratio was used for quantitation. Quality assurance samples were prepared from stock solutions obtained from High Purity Standards, Charleston, SC. Different concentrations were spiked into serum samples and these were assayed over several months to obtain target values. All samples were tested in kinetic energy discrimination (KED) mode, as the method used also measures iron. All quality assurance samples had adequate recovery and the internal standard counts were stable throughout the run. The r^2^ for the calibration curve for all runs was >0.990.

### Preparation of human monocyte-derived macrophages (MDM) and loading with drug-containing nanoparticles

Human monocytes were purchased from the UNMC Department of Pharmacology cell core facility. The monocytes were purified by counter-current centrifugal elutriation, as previously reported.[23] The differentiation of monocytes was carried out as described before.[24] Briefly, monocytes (7.5 x 10^5^ cells/well/mL in a tissue treated 24 well plate) were incubated in DMEM supplemented with 10% heat-inactivated pooled human serum (Innovative Biologics, Herndon, VA, USA), 10 ng/mL MCSF, 50 μg/mL gentamicin (to prevent replication of extracellular bacteria), 10 mM HEPES (pH 7.0), and 10 mM sodium pyruvate at 37°C in 5% CO_2_ humidified atmosphere. Half of the media was replaced on the 5^th^ day of incubation and then every 2 days thereafter, a complete media change occurred until day 10 of incubation. At the 10^th^ day, the differentiated MDMs were treated with DMEM supplemented with 1% human serum. On day 11, MDMs were incubated with drug (GaTP or rifampin, 300 μM) or nanoparticle (300 μM) for 24 h at 37°C in 5% CO_2_ humidified atmosphere. The nanoparticle loaded-MDMs were washed with PBS and incubated for up to an additional 5 to 15 days prior to infection.

### Determination of *M*.*tb* growth residing in macrophages

For pretreatment experiments, drug (300 μM Ga nanoparticle)[20, 24] treated or control (no drug or nanoparticle treatment) MDMs were infected with *M*.*tb* (H37Rv, MOI = 0.2) for 24 h, at days 5, 10 or 15 following drug or nanoparticle treatment (using media without gentamicin). After infections, MDMs were washed with PBS to remove extracellular *M*.*tb* and then placed in media containing gentamicin. MDM were then lysed 2 days after infection for determination of MDM-associated CFU. Briefly, after removing the media from the wells, iced sterile water (300 μL) was added to the wells followed by incubation on ice for 10 min. The MDM were treated with 1.2 mL of lysis buffer containing 55% of 7H9 broth, 20% of the 0.25% SDS and 25% of the 20% BSA in PBS. The lysed cells were centrifuged at 14,000 × g for 15 min and the pellets were resuspended, serially diluted in sterile PBS and plated onto 7H11 agar plates with colonies counted 2–3 weeks later. For treatment post-*M*.*tb* infection experiments, THP-1 macrophages were infected with H37Ra (MOI = 1) for 1 h and incubated with the drugs for 24 h. After washing with PBS to remove drugs, the macrophages were incubated in RPMI 1640 containing 1% FBS and 50 μg/mL gentamicin. The macrophages were lysed to determine CFU at day 3 and 6 post-infection as described above.

### Synthesis of fluorescein-conjugated Ga(III) or rifampin nanoparticles

Fluoresceinamine was used to prepare dye-labeled nanoparticles as previously described.[20] In brief, DCC and N-hydroxysuccinimide (NHS) was added at room temperature to a solution of poly(lactic-co-glycolic acid) (PLGA) in dichloromethane. The mixture was stirred overnight at rt. After activating the carboxylic acid, fluoresceinamine in DMSO was added to synthesize fluorescein-labeled PLGA. The labeled PLGA was mixed with non-labeled PLGA in 1:3 ratio. Fluorescent Ga nanoparticles were prepared using single emulsion technique. To a PLGA solution in CH_2_Cl_2_ was added Ga(III) meso teteraphenyl porphyrine chloride or rifampin. This organic solution was slowly added to 1% polyvinyl alcohol cooled in an ice, and sonicated at 20% amplitude for 10 min. The suspension was stirred overnight to remove CH_2_Cl_2_ and centrifuged at 12,000 × g for 20 min. The obtained pellet was washed with water three times by centrifugation at 12,000 × g for 20 min. Finally, the pellet was resuspended in deionized water, lyophilized and stored at -80°C until needed.

### Subcellular nanoparticle localization and immunocytochemistry analysis

Monocytes (5 x 10^4^ cells/well) were differentiated to MDMs in 8-chamber plates (Lab-Tek^®^, U.S.A) as described above. After differentiation, MDM were treated with GaTP, GaTP nanoparticles (GaNP), or fluorescein-conjugated PLGA for 24 h at 37°C, followed by fixation with 4% phosphate-buffered paraformaldehyde for 30 min. Permeabilization was performed in 0.1% Triton X-100 in PBS at rt for 10 min. After blocking with 5% BSA in PBS for 30 min, the slides were incubated with rabbit polyclonal Cathepsin D antibody (Millipore, USA) and rabbit polyclonal Galectin 3 antibody (Thermo Scientific, USA). Visualization of immunostaining was done incubating with Alexa Fluor^®^488 goat-anti-rabbit IgG (H+L) (Molecular Probes^™^). ProLong^®^ Gold antifade reagent with DAPI was also added to wells.

### Subcellular co-localization of FITC-labeled *M*.*tb* (H37Ra) with LysoTracker in THP-1 macrophages

The *M*.*tb* strain, H37Ra, was tagged with fluorescin isothiocyanate (FITC). The strain was grown to OD_600_ nm of 0.4 in 7H9 medium supplemented with OADC (10%) and washed with 7H9 medium by centrifuging at 10000 × g for 15 min. The pellet was resuspended in 7H9 medium containing 100 μg/mL FITC and incubated at room temperature for 30 min. The FITC-labeled H37Ra was pelleted, washed with PBS three times and resuspended in RPMI 1640 supplemented with 1% FBS. GaNP-treated or untreated THP-1 macrophages were prepared and seeded on glass coverslips as described above and infected with the FITC-labeled H37Ra for 4 hours. The cells were washed with PBS three times and incubated overnight in the medium containing 50 μg/mL gentamicin. After washing with PBS twice, the cells were incubated with 100 nM LysoTracker Red DND-99 (Invitrogen Life Technologies, CA, USA) in RPMI 1640 supplemented 1% FBS for 1 hour followed by fixation with 4% paraformaldehyde in PBS at room temperature for 30 min. Coverslips were mounted by ProLong^®^ Gold anti-fade reagent with DAPI. Confocal images were obtained using a Zeiss LSM 710 microscope. Percentage of FITC-labeled *M*.*tb* co-localized with LysoTracker was determined by analyzing 40 ~ 70 cells.

### Whole cell extracts and Western blot analysis

MDMs cultured in 6-well plates were washed with ice-cold PBS buffer and cell proteins were extracted in Ripa buffer containing Halt^™^ protease inhibitor cocktail (Pierce Biotechnology, IL, USA) and 10 mM DTT. The lysates were collected and centrifuged at 4°C at 12,000 × g for 10 min. The supernatants were collected and the BCA assay was performed to determine protein concentration. Protein samples were electrophoresed under denaturing conditions using 4–20% polyacrylamide gels (Bio-Rad, USA). After transferring to a PVDF membrane, the membrane was analyzed with rabbit polyclonal Cathepsin D antibody (Millipore, USA) and rabbit monoclonal Galectin 3 antibody (Thermo Scientific, USA). The bound antibody was detected using horseradish peroxidase-conjugated goat anti-rabbit IgG (Novex, MD, USA) and developed with a chemiluminescent substrate (Pierce Biotechnology, IL, USA). Image J was used for densitometry analysis of membrane blots.

### Statistical analysis

All experiments were performed in triplicate, and data represent the mean ± standard error mean of triplicate samples (n = 3). Statistically significant variance for collected data was determined by Two-way ANOVA or Student’s *t* test.

## Results

### Preparation and characterization of nanoparticles

**Fig 1:**
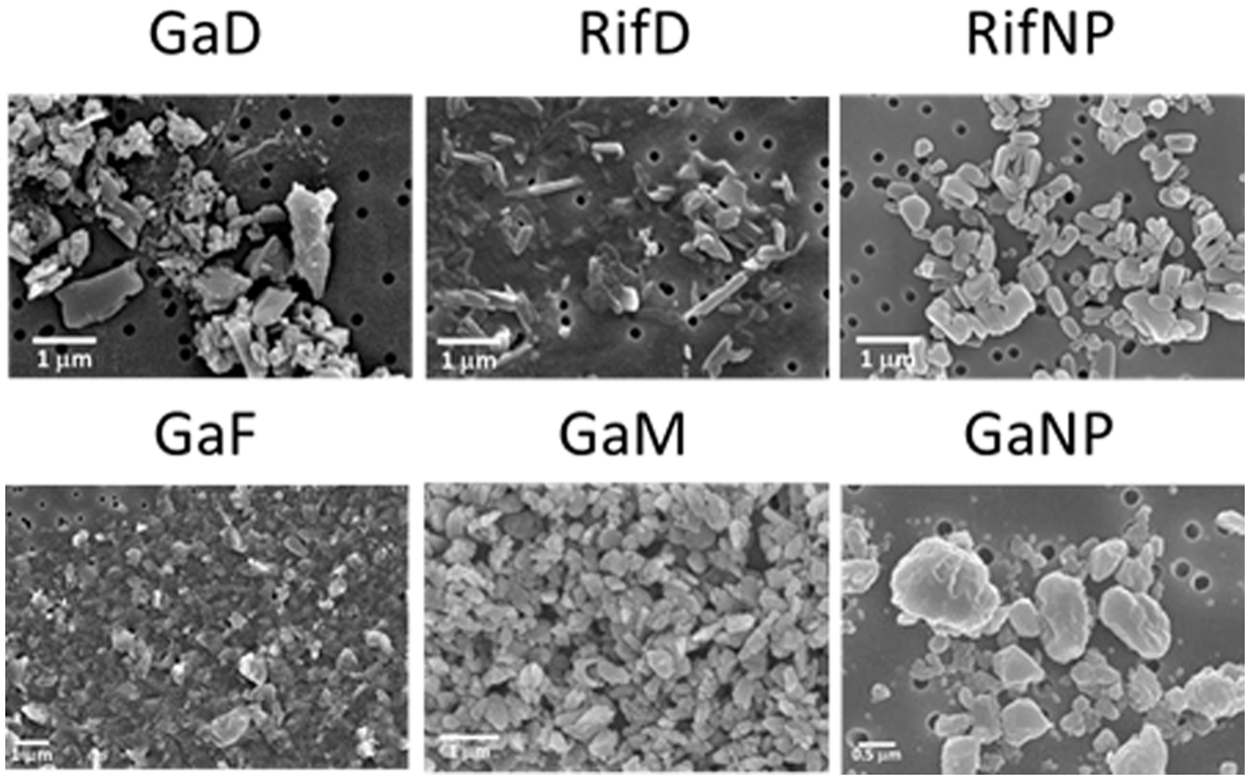
SEM images of nanoparticles containing Ga(III) or rifampin. Nanoparticle morphologies are different depending upon the drug encapsulated. GaD is a rough rectangular shape, while RifD is a long rod shape. GaM shows aggregation of particles. GaF, GaM, GaNP and RifNP are rectangular, with different sizes. GaTP: Gallium(III) *meso* tetraphenylporphyrine chloride; GaNP: nanoparticle encapsulating GaTP; GaF: folate-conjugated nanoparticles encapsulating GaTP; GaM: mannose-conjugated nanoparticles encapsulating GaTP; RifD: dendrimer nanoparticle encapsulating rifampin; and RifNP: P118 nanoparticle encapsulating rifampin.

Mannose- or folate-conjugated F127 were synthesized by procedures outlined in S1 Fig. Mannose and folate groups were introduced to target the respective mannose and folate receptors on macrophages, with the goal of improving cell uptake and retention of drug content within cells. Nanoparticles were prepared from the mixture of free drug (Ga(III) or rifampin) and polymer (F127, P188 poloxamer, mannose/folate-conjugated F127 or dendrimer) by a high pressure homogenization technique. All of the nanoparticles manufactured differed by size, polydispersity index (PDI) and ζ-potential (Table 1). Relatively larger nanoparticles (698 nm for rifampin dendrimer nanoparticle (RifD) and 882 nm for gallium dendrimer nanoparticle (GaD)) were obtained when dendrimer was used. Smaller nanoparticles were manufactured using poloxamer F127 and P188. The measured ζ-potentials ranged from +35 for GaNP to -35.3 mV for rifampin nanoparticles (RifD). All nanoparticles manufactured with F127 polymer are positively charged, whereas dendrimer and rifampin nanoparticles are negatively charged. These positively charged nanoparticles that are made of block polymer should provide an advantage by penetrating cells better than anionic nanoparticles.[25] The physical morphology of the nanoparticles was visualized using scanning electron microscopy (SEM), which showed their shapes were dependent on the drug encapsulated. Morphologies of the two dendrimer nanoparticles, GaD and RifD, were different. RifD was a long rod shape, while GaD was small and rectangular. GaNP, RifNP, Ga mannose (GaM) and Ga folate nanoparticles (GaF) showed a similar rectangular shape (Fig 1), with different sizes than GaD. (Table 1)

**Table 1:** Manufactured nanoparticles.

	Drug	Polymer	Size (nm)	PDI	ζ-potential
GaNP	Ga(III)^a^	F127	305	0.29	+35[20]
GaM	Ga(III)^a^	Mannose-conjugated F127	309	0.4	+17.0
GaF	Ga(III)^a^	Folic acid-conjugated F127	329	0.4	+30.6
GaD	Ga(III)^a^	Dendrimer	882	0.29	-10.8
RifD	Rifampin	Dendrimer	698	0.36	-35.3
RifNP	Rifampin	P188	350	0.19	-10.4

^a^. Ga(III) meso-teteraphenyl porphyrine chloride.

PDI = polydispersity index

### Uptake of nanoparticles by macrophages

**Fig 2:**
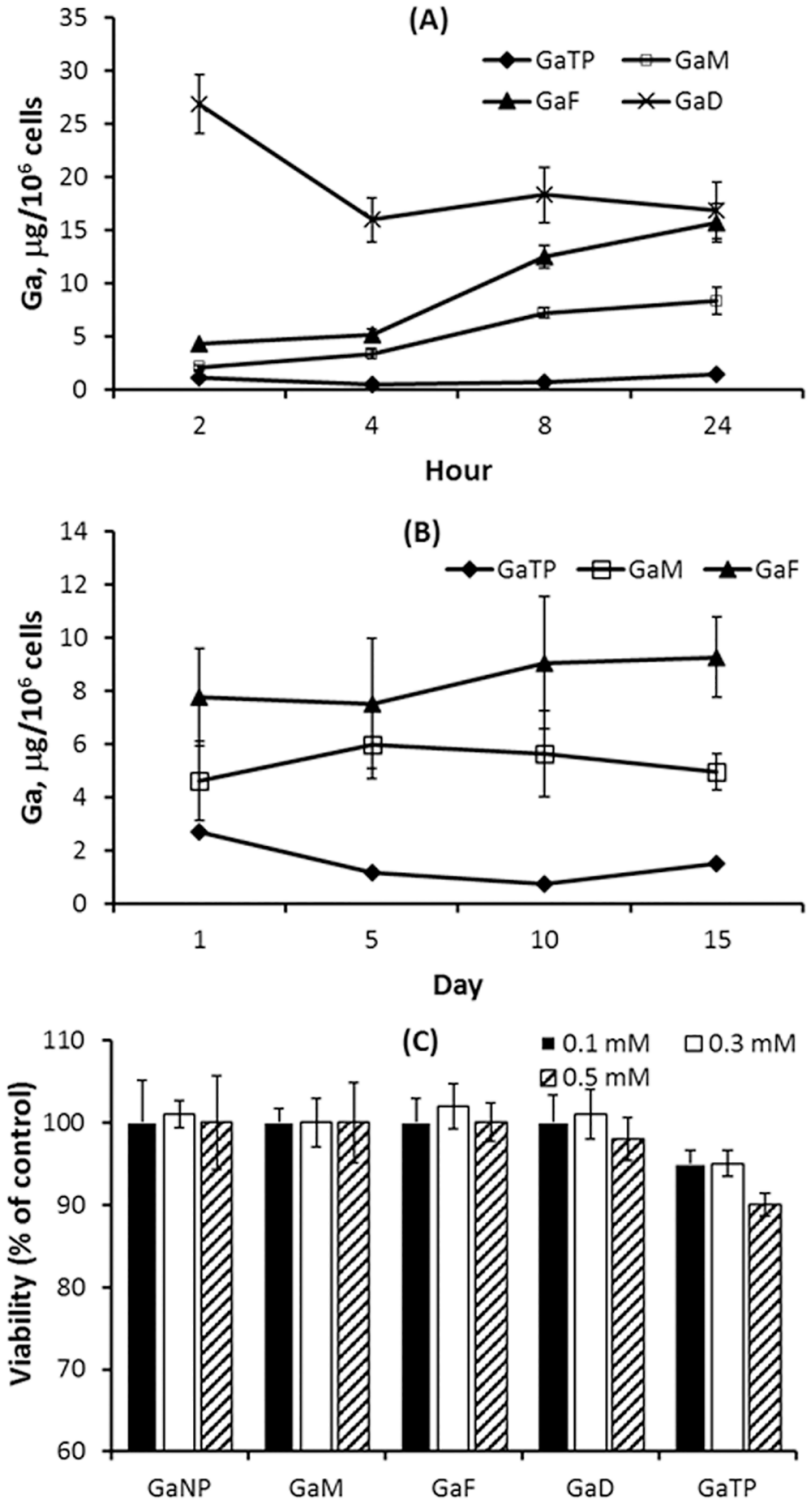
Ga(III) uptake (A), retention (B), and cytotoxicity (C) following incubation of THP-1 macrophages with Ga(III) nanoparticles. (A) Gallium associated with THP-1 cells levels was measured 2, 4, 8 and 24 h following treatment of THP-1 macrophages with Ga(III) nanoparticles. (B) Ga retained in macrophages over time was determined at day 1, 5, 10, and 15 following treatment of THP-1 macrophages with the nanoparticles. THP-1 cells were administered 300 μM nanoparticles containing Ga(III). (C) Cytotoxicity of Ga(III) nanoparticles on THP-1 macrophages as measured by reduction of resazurin. GaTP: gallium(III) *meso* tetraphenylporphyrine chloride GaF: folate-conjugated nanoparticles encapsulating GaTP. GaM: mannose-conjugated nanoparticles encapsulating GaTP.

To determine the macrophage-loading efficacy of nanoparticles targeting the folate or mannose receptor of macrophages, THP-1 cells were treated with GaD, GaTP, GaM or GaF for 1, 4, 8 and 24 h (Fig 2). The amount of Ga(III) taken up by the THP-1 cells was measured by ICP-MS. Ga(III) content was 2.5 and 10 fold higher using GaF compared to GaM and free Ga(III) at 24 hour, respectively. The highest Ga(III) amount was observed after 2 hour using GaD, indicating that GaD was taken up faster than the other NPs by the THP-1 macrophages (Fig 2A). In addition, higher amounts of Ga(III) from nanoparticles were retained in THP-1 cells for up to 15 days, as compared with free drug treated THP-1 cells (Fig 2B). Among the F127 nanoparticles, folate-nanoparticles were taken up much better (Fig 2). However, the retention study (Fig 2B) indicates that folate-conjugated nanoparticles (GaF) release Ga(III) slower than mannose-conjugated nanoparticles (GaM).

### Drug-loaded nanoparticles decrease the growth of *M*.*tb* in macrophages

**Fig 3:**
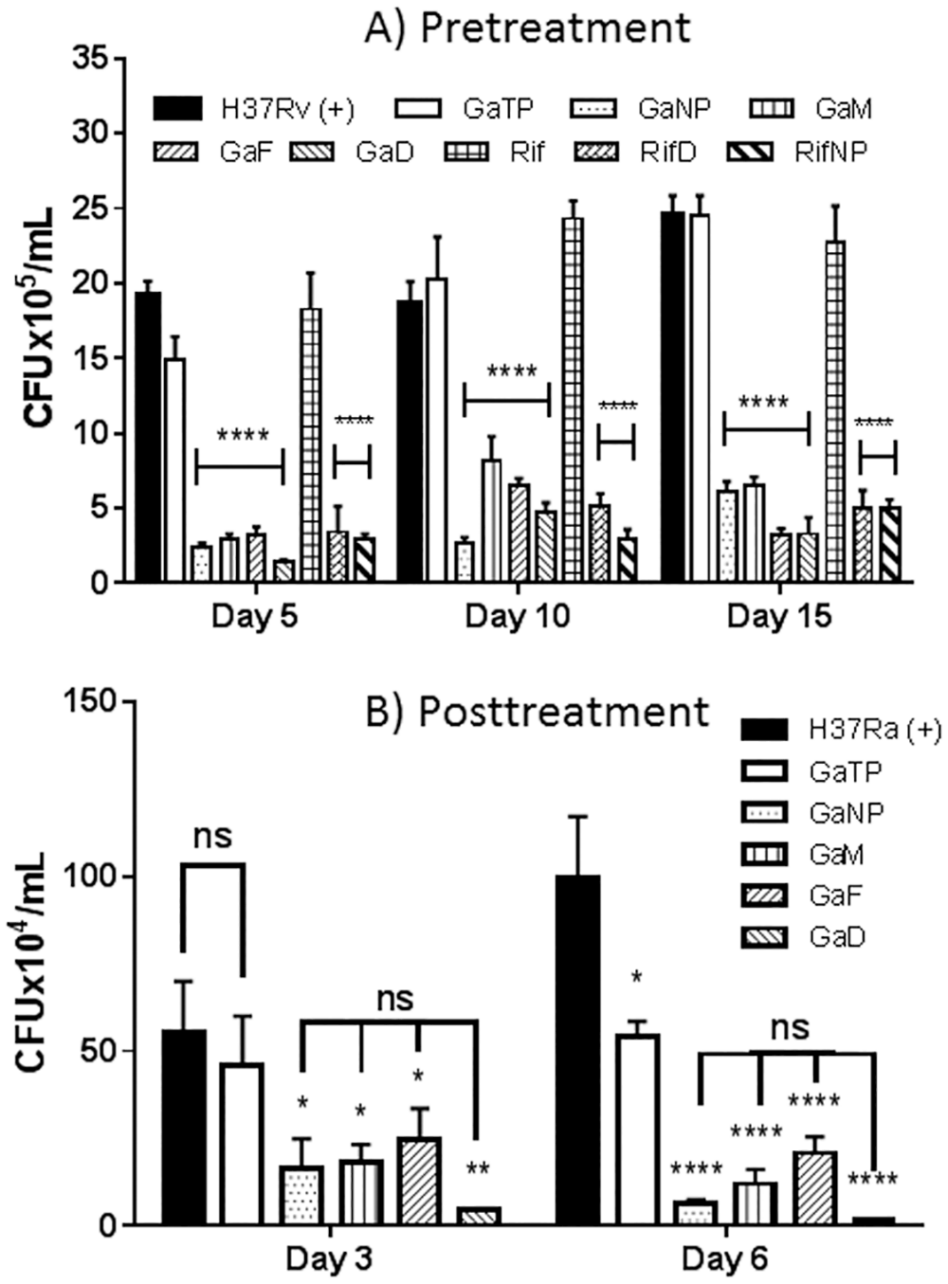
Anti-tuberculous activities of nanoparticles encapsulating Ga(III) or rifampin in *M*.*tb* infected macrophages. (A) Pretreatment: MDMs were infected with *M*.*tb* (H37Rv, MOI = 0.2, 24 h) at days 5, 10, and 15 following MDM treatment with nanoparticles (300 μM Ga) and then cultured for 2 days, following which the MDM were lysed and *M*.*tb* CFU determined. (B) Treatment post-infection: THP-1 macrophages were infected with *M*.*tb* (H37Ra, MOI = 1, 1 h) followed by 24 h treatment with specific nanoparticles. They were then incubated for an additional 3 or 6 days. Following that the MDM were lysed and viable intracellular *M*.*tb* determined by CFU. Data represent the mean ± SEM of triplicate samples (n = 3). Statistical differences were determined using Two-way ANOVA: *p < 0.05, **p < 0.01, ***p < 0.001, ****p < 0.0001 compared with non-drug treated control.

We first determined the minimum inhibitory concentration (MIC) of GaTP against *M*.*tb* strain H37Ra in media (7H10 + OADC, BD BBL^™^ Middlebrook, USA) that contains 40 mg/L ferric ammonium citrate. GaTP exhibited a MIC of 4 μg/mL. Anti-tuberculous activities of nanoparticles for *M*.*tb* within human MDMs or THP-1 macrophages were then evaluated (Fig 3). In order to examine the long acting potential of the nanoparticle for prophylaxis, MDMs were treated with nanoparticles before infection (Fig 3A). All nanoparticles showed significant inhibition of *M*.*tb* for up to 15 days following drug loading of MDMs, suggesting prolonged drug retention and release from the nanoparticles within MDMs. In contrast, the free drug, GaTP, revealed no inhibition on the growth of *M*.*tb* 5 days after incubation of MDMs with the drug. These results are consistent with our prior data with *M*. *smegmatis* and an attenuated strain of *M*.*tb* (H37Ra).[20, 21] Drug uptake and retention studies confirmed prolonged drug release and a high amount of drug uptake by MDMs (Fig 2).

There was no significant difference in inhibition of growth of *M*.*tb* in MDMs between nanoparticles encapsulating Ga(III) or rifampin (Fig 3A). GaNP and GaM inhibitory activities were about 2-fold at day 15 compared to day 5, while GaD and GaF nanoparticles showed consistent inhibition against H37Rv throughout that period. This is likely due to the more sustained release of Ga over time by GaD and GaF nanoparticles. Interestingly, free drug rifampin itself did not show any inhibitory activity at the times studied after MDM drug loading. Rifampin is known to penetrate cells effectively.[24] Hence, these results are probably due to failure of the MDM to retain adequate amounts of rifampin at day 5 and beyond when the drug is presented in this form (Fig 3A).

Next, the inhibitory activities of Ga nanoparticles were tested against *M*.*tb*-infected THP-1 macrophages (Fig 3B). Consistent with our early results with GaNP and GaTP [21], all Ga nanoparticles showed significant growth inhibition of *M*.*tb* in THP-1 macrophages when they were lysed on days 3 and 6 after infection (Fig 3B). These Ga nanoparticles, regardless of their size and zeta potentials, showed 3–10 and 5–20 fold inhibition of *M*.*tb* growth compared to (+) control at days 3 and 6, respectively. Similar to pretreatment experiments (Fig 3A), the dendrimer nanoparticle GaD showed the most promising anti-tuberculosis activity, in spite of its larger relative size. These results support the concept that Ga nanoparticles are promising anti-tuberculous agents for inhibition of the intracellular growth of *M*.*tb*.

### Co-localization of nanoparticles and virulent *M*.*tb* within MDM

**Fig 4:**
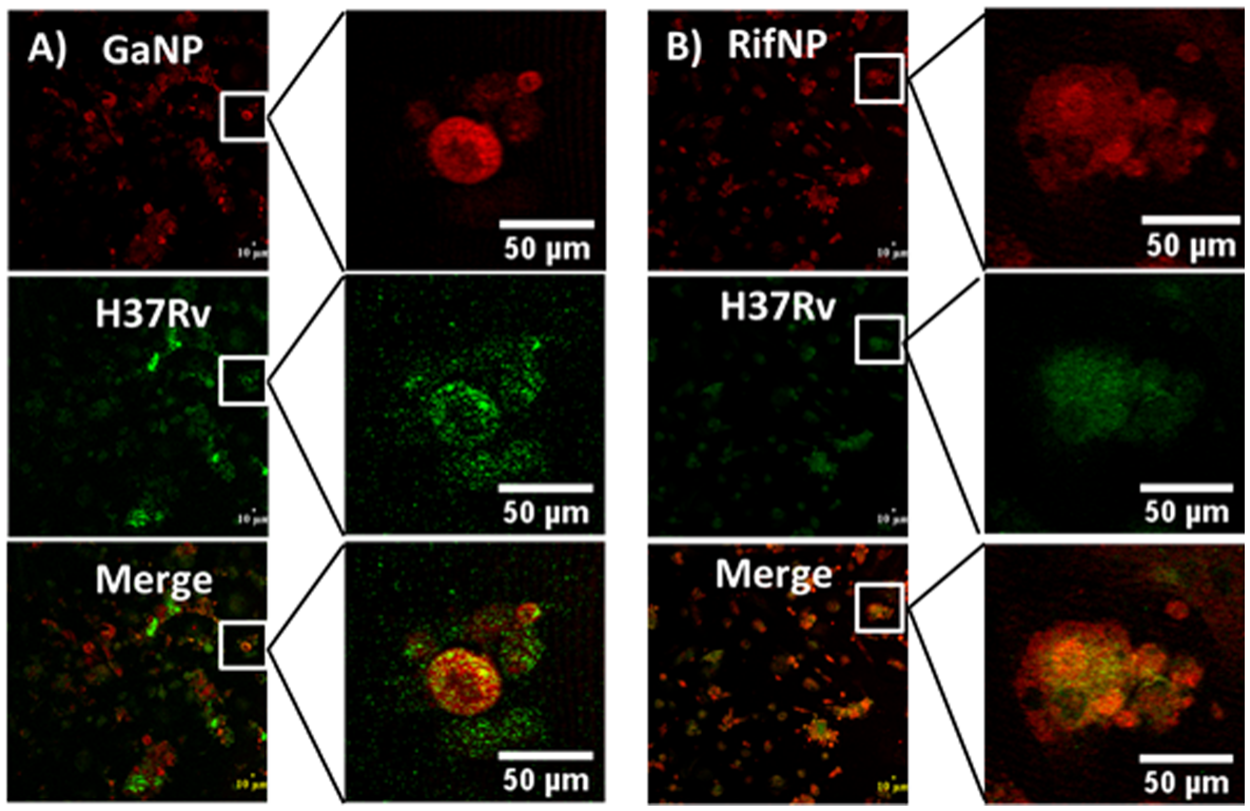
Co-localization of *M*.*tb* (H37Rv) and nanoparticles within MDMs. MDMs were treated with the fluorescein-conjugated nanoparticles (PLGA-GaNP and PLGA-RifNP, Red) for 24 h before infection with *M*.*tb* H37Rv expressing green fluorescent protein (GFP). The relative locations of the nanoparticles and *M*.*tb* were determined by confocal microscopy.

Fluorescein-conjugated nanoparticles and *M*.*tb* reside in the same macrophage.[26, 27] However, in order to determine the distance between both, fluorescein-conjugated nanoparticles and virulent *M*.*tb* expressing GFP within MDM, confocal microscopy experiments were performed. The confocal photomicrographs showed that nanoparticles and virulent *M*.*tb* co-localized (Fig 4). No difference was detected between RifNP and GaNP treated MDMs. Both RifNP and GaNP exhibited ~ 90% co-localization with phagosome containing *M*.*tb* within MDMs (S5 Fig), consistent with fusion between the *M*.*tb*-containing phagosome and endosome-containing nanoparticles.

### Macrophages infected with *M*.*tb* and treated with GaNP increase the expressions of galectin 3 and cathepsin D

**Fig 5:**
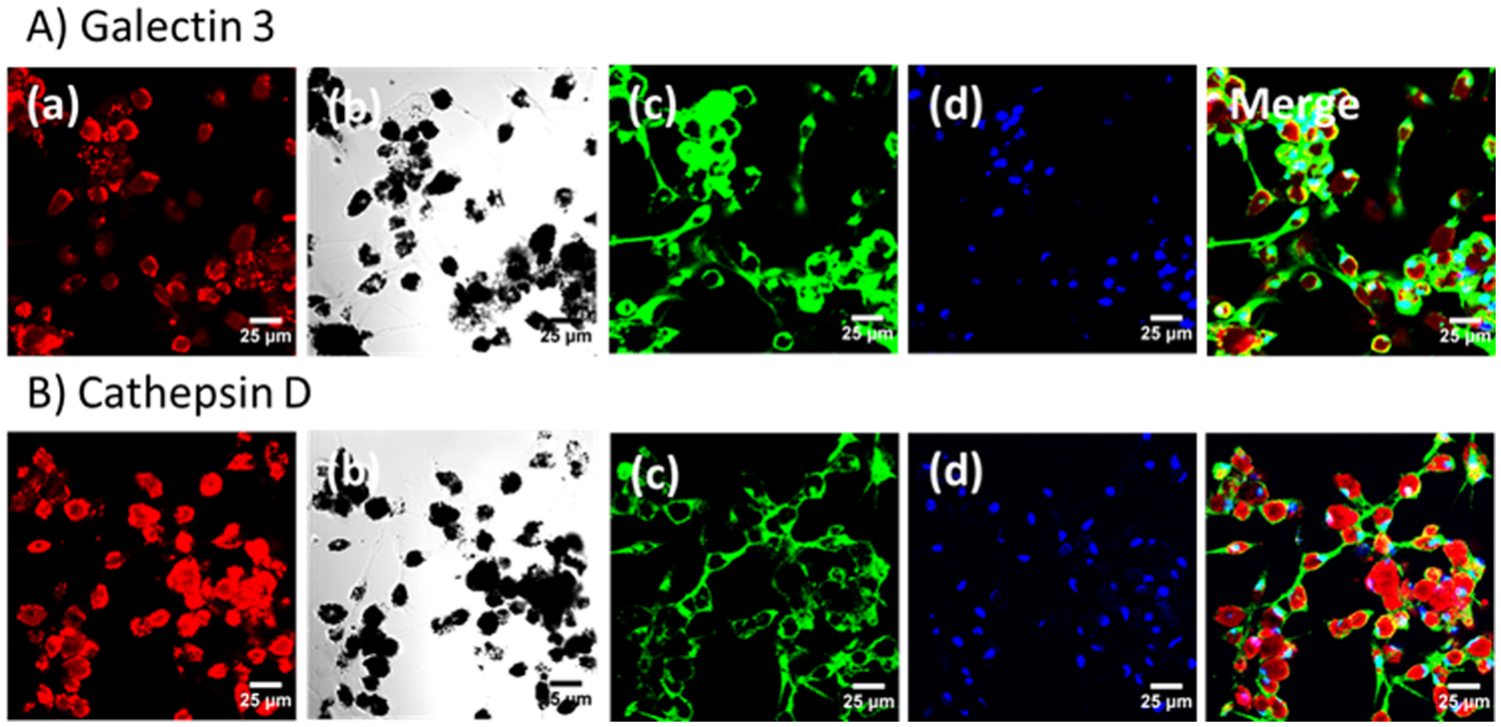
Confocal images of macrophages treated with GaNP. a) Fluorescein-conjugated GaNP (Red), b) macrophages and GaNP, c) galectin 3 (A) or cathepsin D (B), d) Nuclei (Blue). Nanoparticle-treated MDMs were incubated with primary rabbit galectin 3 or cathepsin D. These proteins were visualized by incubation with Alexa Fluor^®^488 goat-anti-rabbit antibody. The scale bar represents 25 μm.

**Fig 6:**
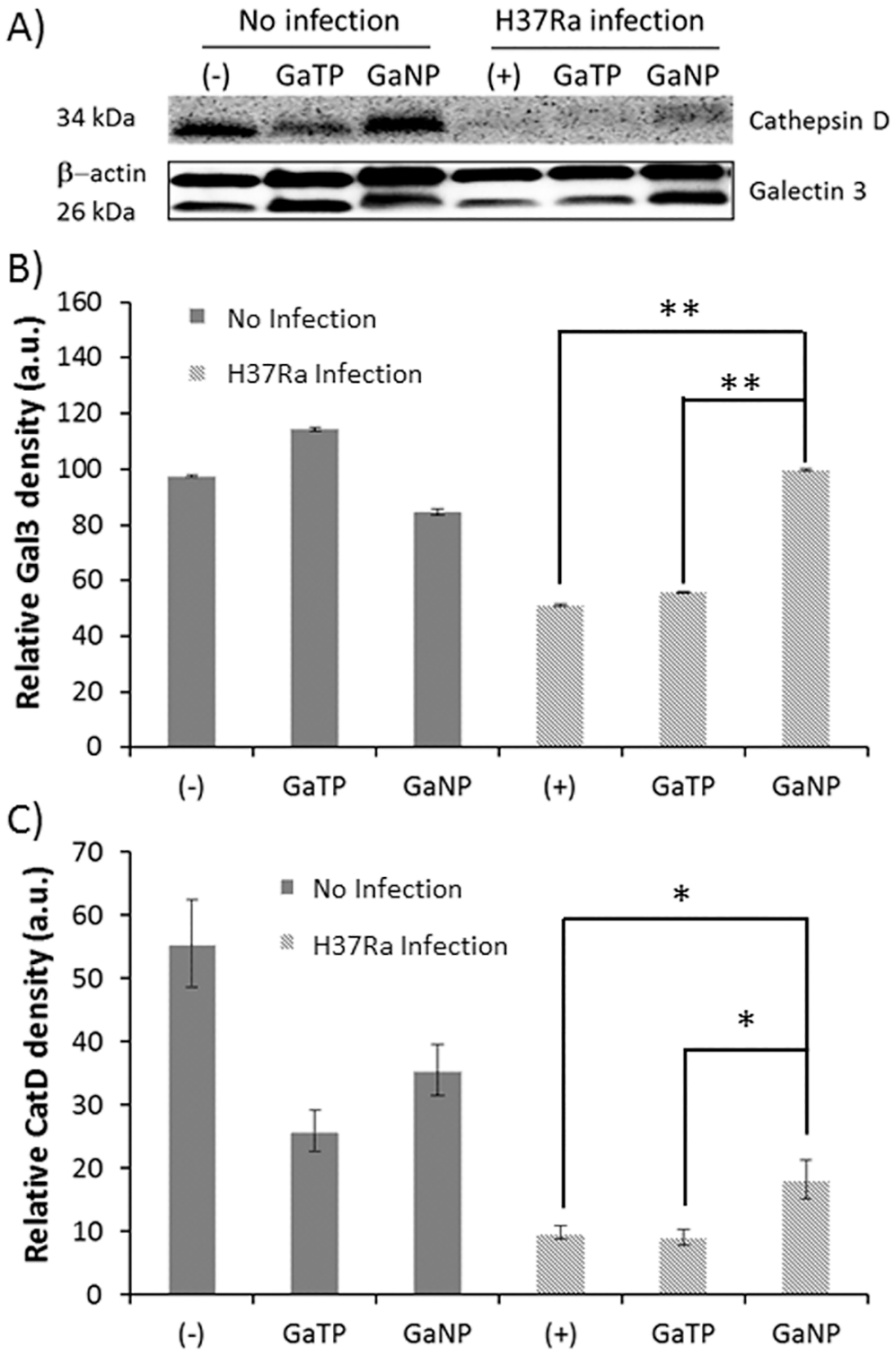
Expression of cathepsin D and galectin 3 in MDMs. MDMs treated with drugs were infected with H37Ra for 4 h (MOI = 1), following which the cells were lysed and levels of cathepsin D and galectin 3 were determined by immunoblot and quantitated by densitometry. Data represent the mean ± SEM of triplicate samples (n = 3). Statistical differences were determined using Student’s *t* test: *p < 0.05, **p<0.01 compared with non-drug treated (+) control.

Fluorescein-conjugated PLGA nanoparticles containing Ga(III) were prepared in order to determine their location within MDM. Confocal images clearly indicate that treatment of MDMs with nanoparticles does not cause degradation of the Ga nanoparticles and confirms their location within the MDMs (Fig 5a and 5b). Two proteins, galectin 3 (Gal3) and cathepsin D (CatD), important in macrophage phagosome maturation were visualized, indicating the presence of phagosomes within the MDMs (Fig 5).[28] We observed co-localization of the Ga nanoparticles and Gal3 (Fig 5A). Similarly, the co-localization of CatD and Ga nanoparticles was also observed (Fig 5B, S3 and S4 Figs). In the case of CatD, the confocal image is not able to distinguish mature CatD (34 kDa) from its precursors. In order to further understand how phagocytosis is affected by *M*.*tb* and Ga nanoparticles, western immunoblot analysis was carried out to monitor the level of expressions of CatD and Gal3. The negative control (non-infected, first lane in Fig 6) expressed mature CatD, as well as Gal3. GaTP or Ga nanoparticle-treated MDMs (no infection, Lane 2 and 3 in Fig 6A) produced similar Gal3 compared to the negative control. Whereas, a form of mature CatD was reduced in both MDMs. Infection with H37Ra appeared to interrupt CatD maturation. However, the mature CatD was still detectable 4h after infection, while further incubation of infected MDMs stopped maturation of pro-CatD (lane 4 in Fig 6). All MDMs infected with *M*.*tb* H37Ra exhibited a significant decrease in expression of CatD regardless of treatments, but the Ga nanoparticle treated MDMs showed a slightly higher expression of CatD compared to H37Ra infected MDMs (Fig 6C). Likewise, expression of Gal3 decreased when MDMs were infected with H37Ra (lane 4 in Fig 6A and 6B). However, infection of Ga nanoparticle-treated MDMs did not decrease the expression of Gal3 (lane 6, Fig 6B).

### GaNP enhances phagosome maturation inhibited by *M*.*tb*

**Fig 7:**
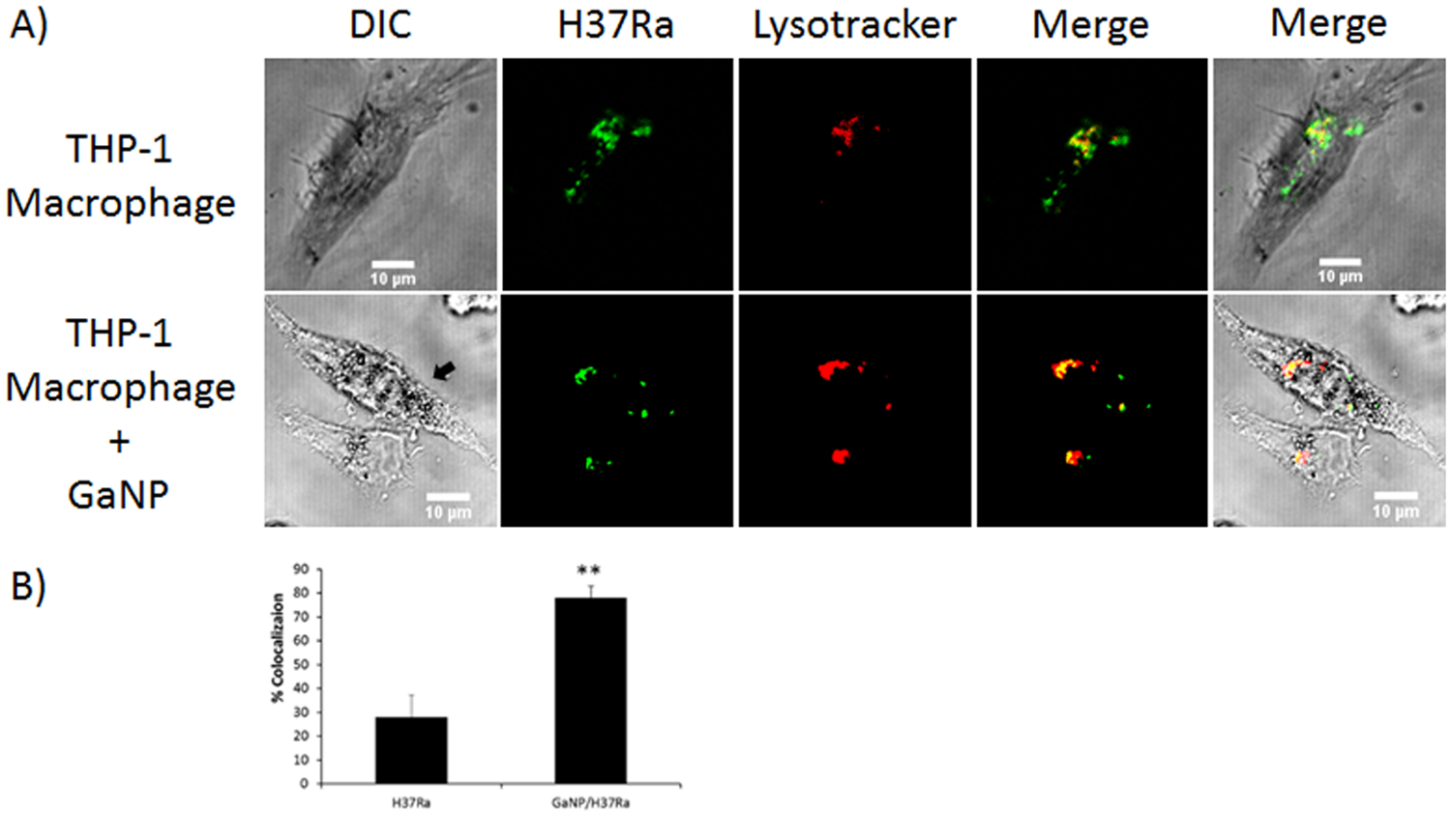
Confocal microscopy assessment of the effect of Ga nanoparticles on phagolysosome fusion in THP-1 macrophages infected with *M*.*tb* H37Ra. **A**) Co-localization of LysoTracker Red DND-99 and FITC-labeled H37Ra. B) Percentage of FITC-labeled bacteria co-localized with acidic compartments labeled with LysoTracker in control and Ga nanoparticle treated MDM. Data are the mean ± SEM determined by analyzing 40 ~ 70 cells. Statistically significant differences were determined using Student’s *t* test: **p<0.01.

An additional experiment was carried out to support the ability of Ga nanoparticles to limit the ability of *M*.*tb* to interfere with phagosome maturation in THP-1 macrophages. LysoTracker was used to co-localize H37Ra in acidic compartments, including lysosomes. Ga nanoparticle-treated macrophages exhibited a higher co-localization of FITC-labeled H37Ra with LysoTracker (78%), whereas only 28% of H37Ra were co-localized with acidic compartments in macrophages that were not treated with Ga nanoparticles (Fig 7 and S6 Fig). These observations demonstrate that accumulation of *M*.*tb* in acidic compartments and phagosome maturation is promoted by Ga nanoparticles.

## Discussion

Morbidity and mortality from TB is increasing in large part to the growing problems of *M*.*tb* antibiotic resistance (XDR and MDR) and HIV-*M*.*tb* co-infection. *M*.*tb*, successfully parasitizes alveolar macrophages, in part by its ability to limit phagosome maturation and grow/replicate in this normally microbicidal environment. In order to survive within the macrophage phagosome, *M*.*tb* must be able to acquire key nutrients. Iron is essential for metabolism of pathogenic bacteria and their virulence in humans.[18, 29] Gallium has been shown to inhibit iron acquisition and metabolism of a number of pathogenic bacterial species and shown efficacy in murine models of infection with these organisms.[14, 15, 18, 19, 30] Gallium nitrate, a FDA approved drug for treatment of hypercalcemia of malignancy, inhibits growth of *M*.*tb* (Erdman, H37Ra, and an isoniazid and rifampin resistant isolate) and/or *M*. *avium* complex (MAC) within macrophages by blocking iron acquisition of intracellular mycobacteria.[13] We have also demonstrated that nanoformulations of Ga potently inhibit growth of *M*. *smegmatis*, and an attenuated strain of *M*.*tb* (H37Ra) within human macrophages, as well as replication of HIV in co-infected macrophages.[20]

Antibiotics incorporated into long acting nanoparticles possess great potential as antimicrobial agents because of their ability for prolonged drug release and targeting to the site of infection using targeted-polymer design.[24] From our literature search, nanoparticles containing Ga (III) and poloxamer have not been tested against a fully virulent strain of *M*.*tb* strain. Thus, six different nanoparticles, including target receptor ligand tagged nanoparticles, were prepared and compared for macrophage targeted delivery and their ability to inhibit growth of the fully virulent *M*.*tb* strain H37Rv, as well as the attenuated H37Ra strain. Dendrimer or F127 block polymers conjugated with mannose or folic acid were used to create antimicrobial nanoparticles containing Ga(III) or rifampin. Various sizes and shapes of nanoparticles with different ζ-potentials were obtained (Table 1), which displayed similar inhibitory activities against the growth of virulent H37Rv residing within MDMs for up to 15 days after macrophage incubation with the nanoparticles (Fig 1). Dendrimers generated bigger nanoparticles, with a range of 691~800 nm, whereas poloxamers generated nanoparticles that were smaller in size, ~300 nm. Dendrimers are a new class of polymers with a highly bio-degradable property and they are non-toxic.[31–33] All synthesized nanoparticles, at concentrations ranging from 100–500 μM, did not induce any detectable cytotoxicity in THP-1 macrophages. The free GaTP-treated cells displayed 95 and 90% viability at 300 and 500 μM, respectively. In general, cellular uptake and cytotoxicity of nanoparticles depends upon surface charge, size, hydrophobicity and shape of the nanoparticles. Surface properties play crucial roles in nanoparticle-cell interaction, which affects cell penetration, as well as cytotoxicity. In our study, we modified the particle surface with folic acid or mannose to improve nanoparticle uptake by macrophages through engagement of cell membrane receptors specific for these molecules. Folic acid conjugated GaF showed higher cellular uptake than mannose-conjugated GaM, probably due to higher levels of cell interaction and internalization by the more positively charged GaF (Fig 2A). Macrophages are known to have folic acid receptors, leading to increased folate-conjugated nanoparticle-cell interaction and cellular uptake.[34] Even though use of different polymers and drugs for nanoparticle construction changed their physical properties, there was no dramatic change in their ability to inhibit the growth of *M*.*tb*.

Our observations support that inhibiting iron acquisition by mycobacteria and the use of nanoparticle delivery is a promising strategy for reducing pathogenic mycobacteria infections. Indeed, Ga nanoparticles were as effective as rifampin nanoparticles in inhibiting intra-macrophage growth of *M*.*tb* under our experimental conditions. However, it is important to better define the mechanism(s) whereby these formulations exhibit their antimicrobial effect. In addition to direct toxicity for the bacteria, Ga might enhance the antimicrobial mechanisms of the macrophage usually subverted by *M*.*tb*. In phagosome biogenesis, the early phagosome contains markers such as early endosomal antigen 1 (EEA1), Rab5 (a marker for early endosome), and the classic marker, transferrin.[35] A small GTPase of the Rab family, Rab5, is recruited to early phagosome through interactions between early endocytic compartments. Phagosomes containing mycobacteria also contain Rab5.[24]

However, maturation of mycobacterial phagosomes stops between the early and late endosome stage, as the late endosomal marker, Rab7, is not detected upon infection of macrophages with virulent *M*.*tb*. [36, 37] These observations are in good agreement with the levels of expression of the lysosomal aspartic hydrolase, cathepsin D, which is targeted to phagolysosomes, upon infection (Fig 6).

Cathepsin D (CatD), a lysosomal aspartic protease, is synthesized in the form of preprocathepsin D on rough endoplasmic reticulum (ER).[38, 39] It undergoes sequential proteolysis (loss of signal peptide, 20 aa) and two N-linked glycosylations, followed by translocation to the golgi and phosphorylation at position six of mannose (M-6-P). The M-6-P pathway delivers this pro-CatD (52 kDa) to the late endosome and then it undergoes proteolytic maturation steps as follows. First, pro-peptide (44 aa) is removed from pro-CatD to generate an intermediate 48 kDa single chain protein that is cleaved into two mature chain enzymes when CatD has reached lysosomes. These consist of light (14k) and heavy (34k) chains. However, after phagocytosis of pathogenic mycobacteria by macrophages, the normal maturation process of the phagosome is inhibited.[40–42] Consistent with this, we found that MDMs infected with *M*.*tb* exhibited reduced mature CatD (Fig 6).

In recent work, we reported that macrophage phagosomes containing *M*. *smegmatis* fuse with early endosome containing nanocarriers, as reflected by co-localization of fluorescent-conjugated nanoparticles and Rab5.[24] In the present study, we found that Ga nanoparticle-treated MDMs showed greater evidence of phagosome maturation when infected with H37Ra, as evidenced by the presence of mature CatD (Fig 6). MDM infected with H37Ra exhibited reduced levels of Gal3 relative to uninfected MDM. Interestingly, Gal3 expression also increased by 1.5 fold when MDMs previously treated with Ga nanoparticles were infected with *M*.*tb* compared to untreated control MDMs. However, no significant change in the levels of Gal3 expression by the macrophages was observed when they were treated with GaTP or GaNP in the absence of *M*.*tb* infection (Fig 6). Gal3, a β-galactoside-binding lectin (~34 kDa), is known to be involved in cancer progression and metastasis, although its mechanism of action remains undetermined.[43] However, Gal3 is abundant in macrophages, as seen in Fig 6, and highly upregulated when monocytes differentiate into macrophages, whereas downregulation is observed when they differentiate into dendritic cells.[44, 45] Furthermore, a phagosome proteomic study in a mouse macrophage cell line revealed Gal3 as a major component [46] and depletion of Gal3 affects phagocytosis of IgG-opsonized erythrocytes and apoptotic cells in vivo and in vitro.[28] Thus, *M*.*tb* infection of macrophages may downregulate Gal3 expression (Fig 6) to prevent or delay phagocytosis that could enhance growth of *M*.*tb* within the phagosome.

Further, accumulation of *M*.*tb* in acidic compartments was studied by assessing the extent of co-localization of *M*.*tb* and LysoTracker-labeled compartments in *M*.*tb*-infected THP-1 macrophages. As shown in Fig 7, 78% co-localization of H37Ra with LysoTracker was observed when infected macrophages were treated with Ga nanoparticles. However, only 28% of H37Ra co-localized with acidic compartments, such as acidified phagosomes and lysosomes, in macrophages not treated with Ga nanoparticles (Fig 7). These results support the possibility that Ga nanoparticles promote phagosome maturation.

**Fig 8:**
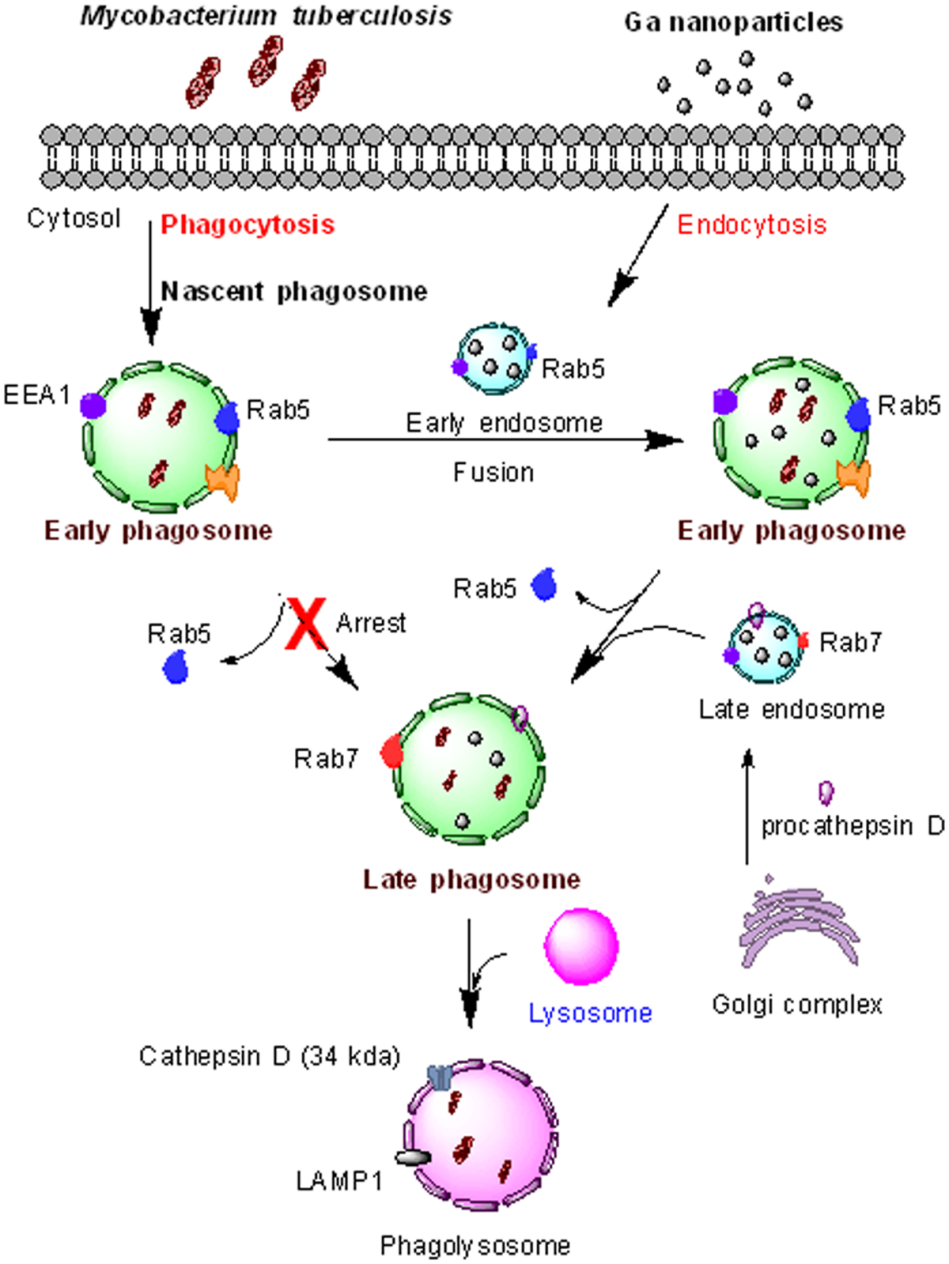
Inhibition of phagosome maturation by *M*.*tb* and restoration by Ga(III) nanoparticles. Early endosome containing Ga nanoparticles fuse with early mycobacterial phagosomes to form a phagosome vacuole containing *M*.*tb* and Ga nanoparticles. EEA1: early endosome antigen 1.

Similar observations were noted in our recent report using *M*. *smegmatis*.[24] Fluorescent-conjugated nanoparticles containing traditional TB drugs or Ga(III) co-localized with *M*. *smegmatis* in phagosomes like Rab5, Rab7, Rab11 and Rab14 and it could be detected. This is consistent with the possibility that the nanoparticles may enhance the maturation process or slow down phagosome (endosome) maturation inhibition by the mycobacteria. It also appears that the Ga nanoparticles may enhance the fusion process between early endosome and mycobacteria-containing vacuoles and facilitate the maturation process of phagosome-lysosome fusion (Fig 8). These results are consistent with previous work in which Ga treatment also appeared to enhance phagosome-lysosome fusion in *M*. *avium*-infected murine bone-marrow-derived macrophages and that iron availability to mycobacteria is required for the organisms’ ability to inhibit this process.[47]

## Conclusion

We have synthesized, characterized and tested six different nanoparticles containing Ga(III) or rifampin. Following ingestion by macrophages, these nanoparticles exhibited sustained drug release over long time periods and significantly inhibited the growth of virulent *M*.*tb* (H37Rv) in MDMs for up to 15 days following drug treatment of the MDM. Likewise, a single treatment of *M*.*tb*-infected macrophages with Ga nanoparticles reduced the growth of *M*.*tb* (H37Ra) for up to 6 days following infection. Targeting macrophages with nanoparticles and ligated nanoparticles for drug delivery showed promising results and warrant future in vivo testing. Changes in the expression of CatD suggest that *M*.*tb* infection impairs pro-CatD trafficking between the Golgi and lysosomes because of depletion of mature CatD. In addition, our observation indicates that Ga nanoparticles are able to interrupt *M*.*tb*-mediated downregulation of Gal3 and thereby the phagosome maturation process, a key component of mycobacterial pathogenesis. Hereby, the synthesized Ga nanoparticles are able to deliver drug to the macrophage, inhibit bacterial growth and reduce the inhibition of phagosome maturation. Studies to identify the exact mechanism whereby the Ga nanoparticles slow or block the ability of mycobacteria to inhibit phagosome maturation are currently in progress.

## Supporting information

S1 Fig **Synthesis of conjugated polymers (A) Synthesis of mannose conjugated F127**. F127 polymer was reacted with succinic anhydride and followed by reaction with mannose. (B) Synthesis of folate conjugated F127: F127 polymer was refluxed with folic acid and purified.
(TIF)

S2 Fig ^1^H NMR spectra of conjugated polymers. (PDF)

S3 Fig Visualizations of galectin 3 and cathepsin D in *M*.*tb* (H37Ra)-infected MDMs. MDMs were incubated with primary rabbit Galectin 3 (A) or Cathepsin D (B). MDMs were infected with H37Ra (MOI = 1) for 4 hours and then fixed with 4% PFA. These proteins were visualized by incubation with Alexa Fluor^®^488 goat-anti-rabbit antibody. Green: Galectin 3 or Cathepsin D, Blue: Nuclei.
(TIF)

S4 Fig Co-localization of *M*.*tb* (H37Ra)-infected MDMs with dyed-nanoparticles and visualizations of galectin 3 and cathepsin D. MDMs were pretreated with fluorescent nanoparticles for 24 h before infection. Red: Fluorescein-conjugated GaNP, Green: Galectin 3 (A) or Cathepsin D (B), Blue: Nuclei. Infected MDMs were incubated with primary rabbit Galectin 3 or Cathepsin D. These proteins were visualized by incubation with Alexa Fluor^®^488 goat-anti-rabbit antibody.
(TIF)

S5 Fig Quantification of co-localization of GaNP with *M*.*tb* (H37Rv) residing in MDMs. (TIF)

S6 Fig Co-localization of *M*.*tb* (H37Ra) and LysoTracker in THP-1 macrophages. (A) THP-1 macrophages were not treated with GaNP, (B) The macrophages were pretreated with GaNP for 24 h before infection. Red: LysoTracker, Green: FITC-labeled H37Ra, Blue: Nuclei.
(TIF)

## Abbreviations

CatD: cathepsin D
DCC: N, N’-dicyclohexylcarbodiimide
DMAP: dimethyl amino pyridine
Ga: gallium (III)
GaD: gallium dendrimer nanoparticle
GaF: Ga folate nanoparticle
Gal3: galectin 3
GaM: Ga mannose nanoparticle
GaNP: GaTP-containing nanoparticle
GaTP: Gallium(III) *meso* tetraphenylporphyrine chloride
KED: kinetic energy discrimination
*M.tb*: *Mycobacterium tuberculosis*
MDM: monocyte-derived macrophages
NHS: N-hydroxy succinimide
PDI: polydispersity index
PLGA: poly(lactic-co-glycolic acid)
RifD: rifampin dendrimer nanoparticle
TB: tuberculosis
TEA: triethyl amine
WHO: World Health Organization
