# Supplementary figures and images for: Gallium nanoparticles facilitate phagosome maturation and inhibit growth of virulent Mycobacterium tuberculosis in macrophages
Source: PLoS One. 2017 May 18;12(5):e0177987. doi: 10.1371/journal.pone.0177987 (PMC5436895; doi:10.1371/journal.pone.0177987)

F127-COOH  
CDCl<sub>3</sub>

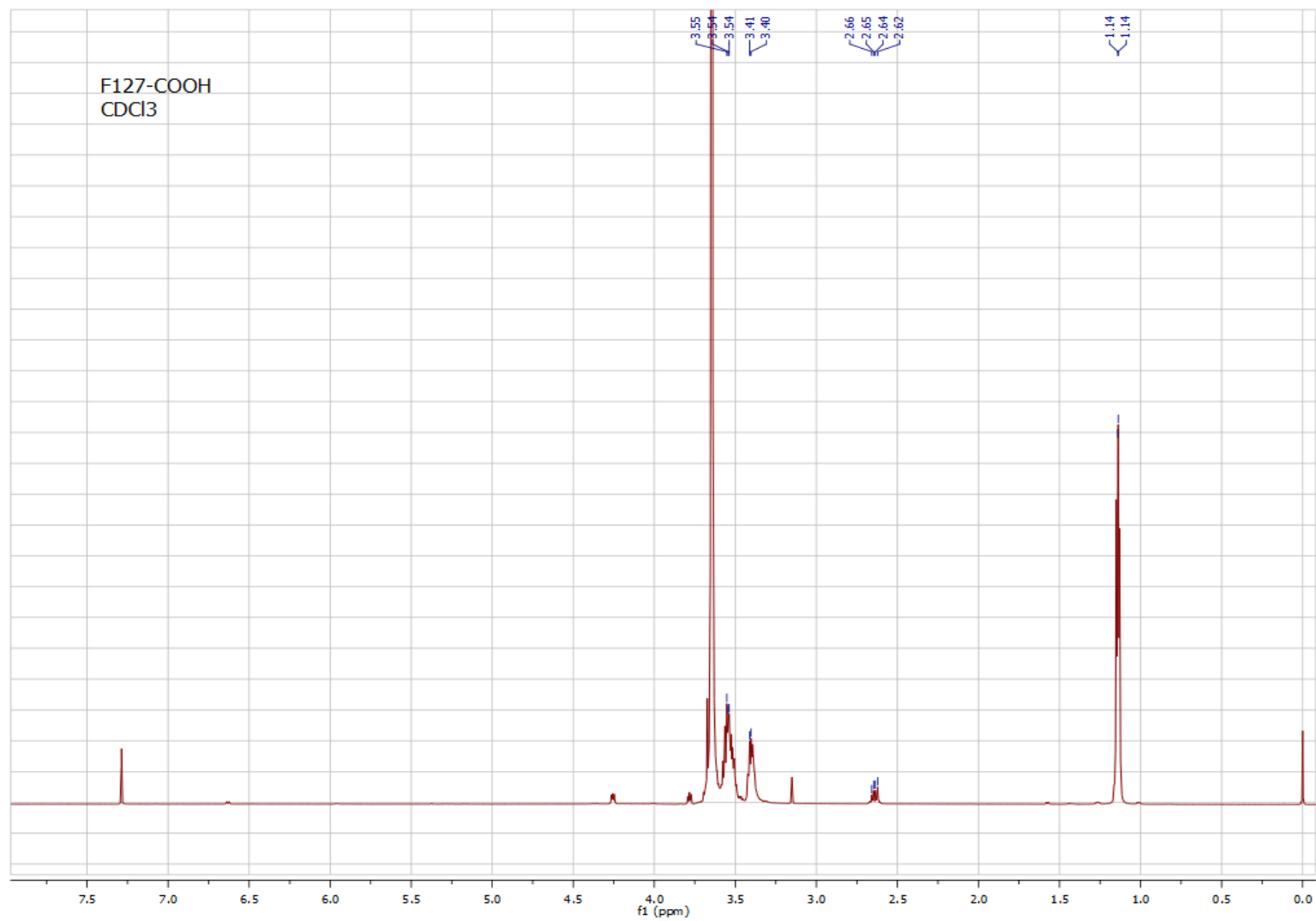

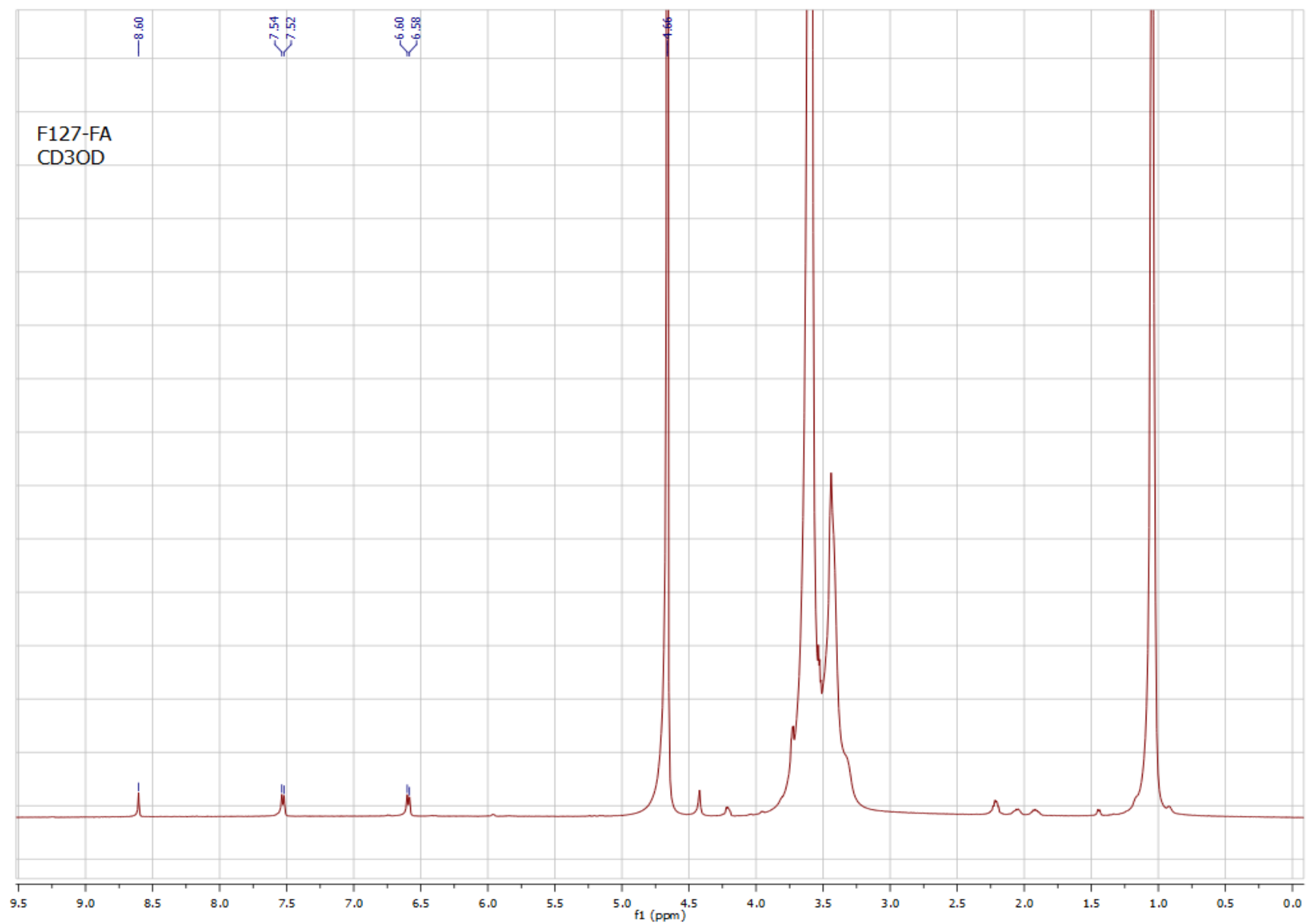

F127-mannose  
DMSO-d6

6.21

5.56

5.55

4.86

4.58

4.39

4.29

4.13

2.79

2.75

2.53

2.49

7.5 7.0 6.5 6.0 5.5 5.0 4.5 4.0 3.5 3.0 2.5 2.0 1.5 1.0 0.5 0.0

f1 (ppm)

Supplement: S2 Fig — (PDF) [file pone.0177987.s002.pdf]

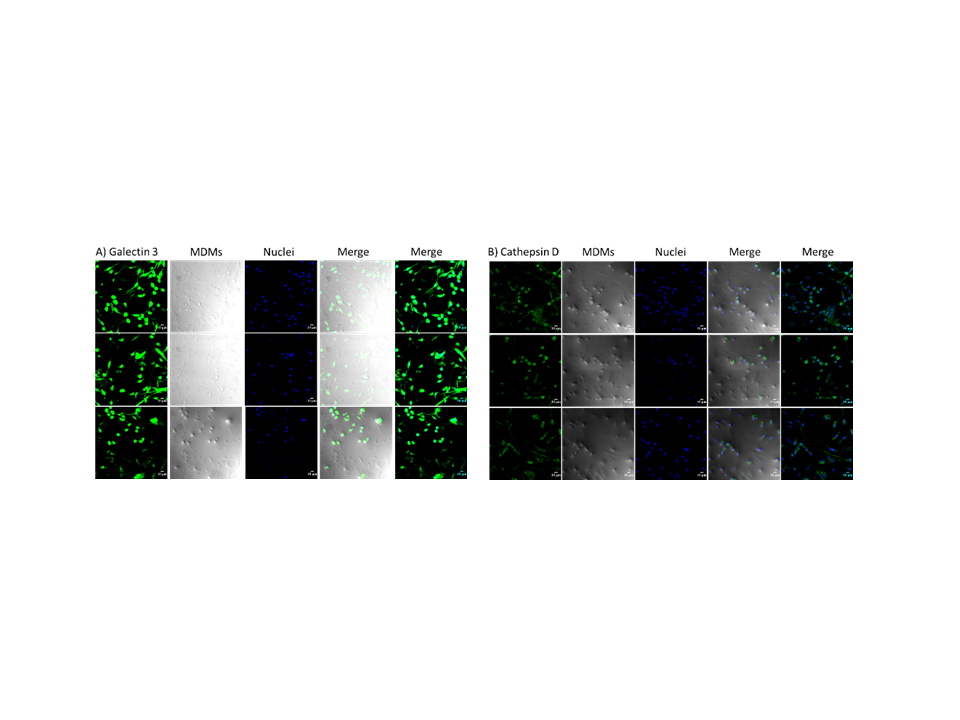

Supplement: S3 Fig — MDMs were incubated with primary rabbit Galectin 3 (A) or Cathepsin D (B). MDMs were infected with H37Ra (MOI = 1) for 4 hours and then fixed with 4% PFA. These proteins were visualized by incubation with Alexa Fluor®488 goat-anti-rabbit antibody. Green: Galectin 3 or Cathepsin D, Blue: Nuclei. (TIF) [file pone.0177987.s003.tif]

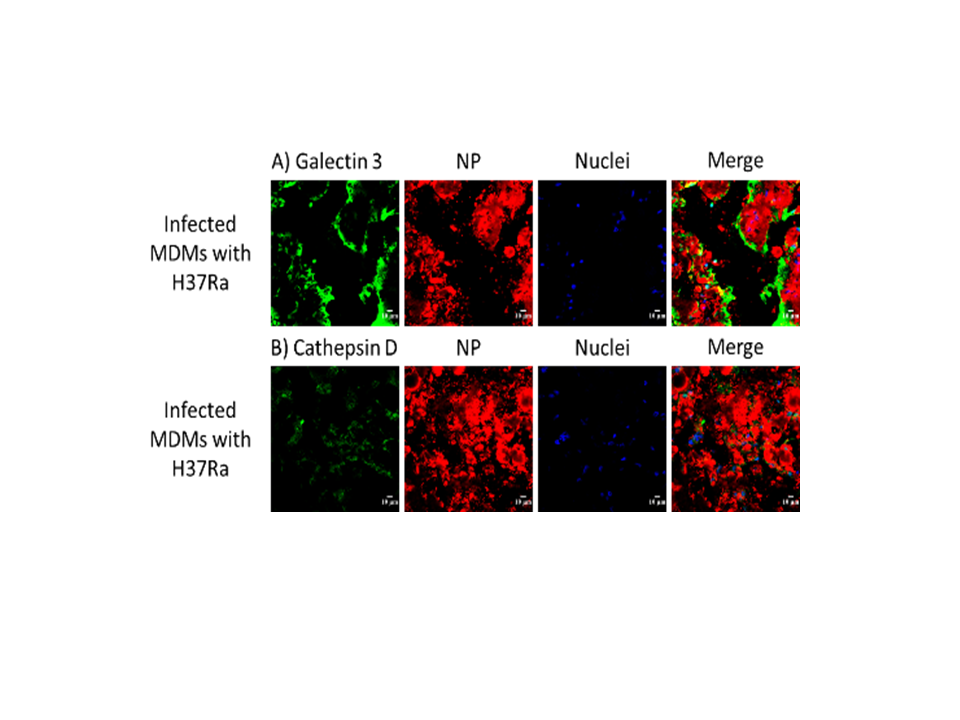

Supplement: S4 Fig — MDMs were pretreated with fluorescent nanoparticles for 24 h before infection. Red: Fluorescein-conjugated GaNP, Green: Galectin 3 (A) or Cathepsin D (B), Blue: Nuclei. Infected MDMs were incubated with primary rabbit Galectin 3 or Cathepsin D. These proteins were visualized by incubation with Alexa Fluor®488 goat-anti-rabbit antibody. (TIF) [file pone.0177987.s004.tif]

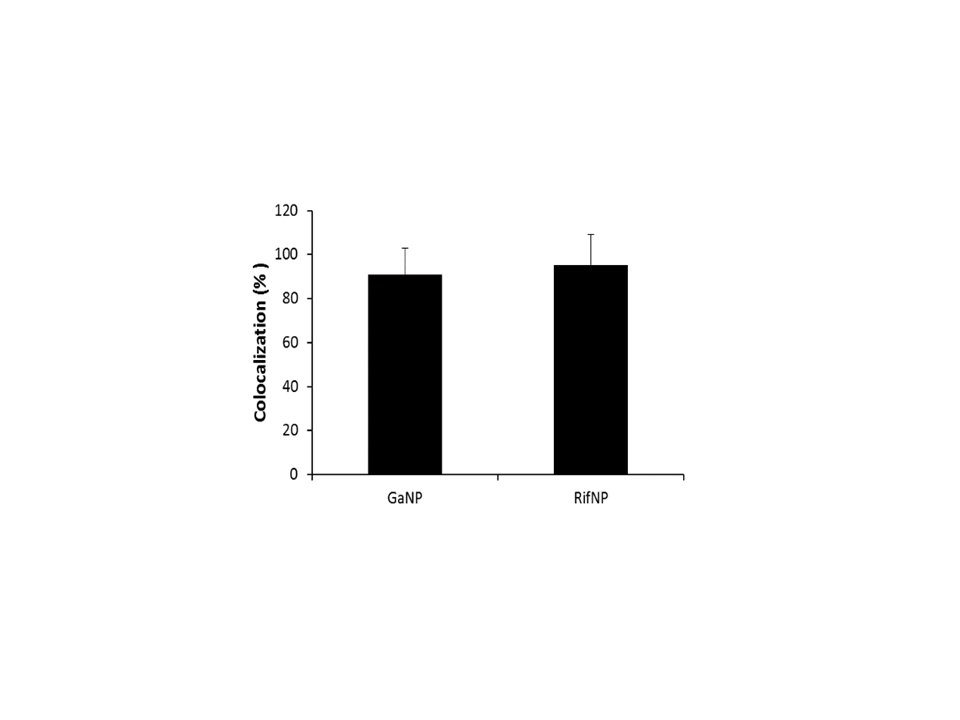

Supplement: S5 Fig — (TIF) [file pone.0177987.s005.tif]

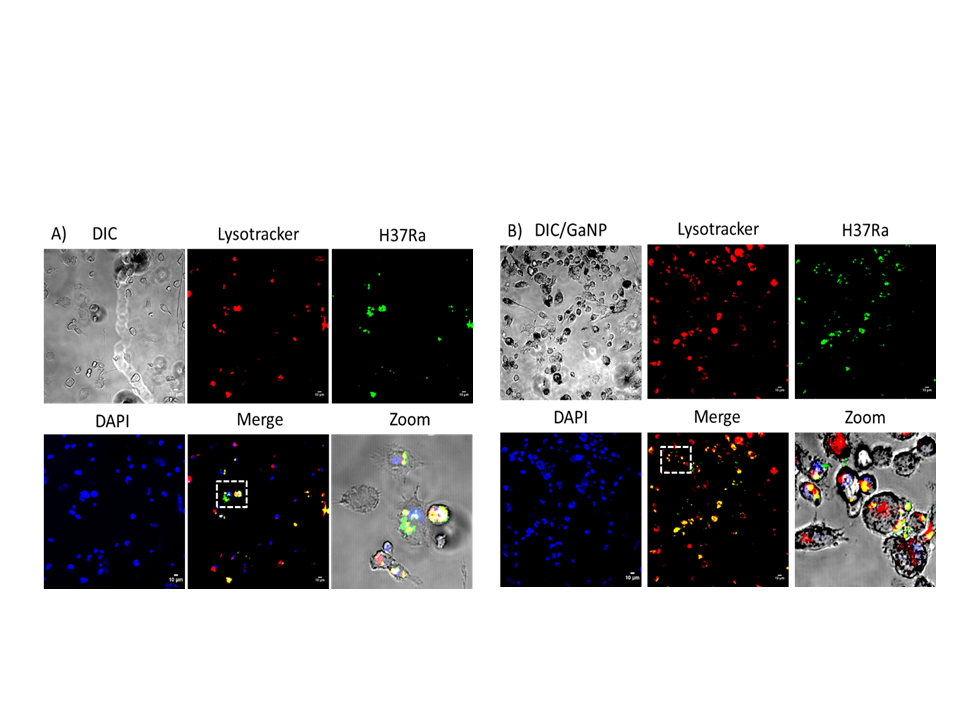

Supplement: S6 Fig — (A) THP-1 macrophages were not treated with GaNP, (B) The macrophages were pretreated with GaNP for 24 h before infection. Red: LysoTracker, Green: FITC-labeled H37Ra, Blue: Nuclei. (TIF) [file pone.0177987.s006.tif]
